# Transcriptomic Evidence of Molecular Mechanisms Underlying the Response of *Lactobacillus plantarum* WCFS1 to Hydroxytyrosol

**DOI:** 10.3390/antiox9050442

**Published:** 2020-05-20

**Authors:** Inés Reverón, Laura Plaza-Vinuesa, Laura Santamaría, Juan Carlos Oliveros, Blanca de las Rivas, Rosario Muñoz, Félix López de Felipe

**Affiliations:** 1 Laboratorio de Biotecnología Bacteriana. Instituto de Ciencia y Tecnología de los Alimentos y Nutrición (ICTAN-CSIC), 28040 Madrid, Spain; inesreveron@gmail.com (I.R.); laura.plaza@ictan.csic.es (L.P.-V.); laura.santamaria.rubio@gmail.com (L.S.); blanca.r@csic.es (B.d.l.R.); rmunoz@ictan.csic.es (R.M.); 2 National Center for Biotechnology (CNB-CSIC), 28049 Madrid, Spain; oliveros@cnb.csic.es

**Keywords:** hydroxytyrosol, *Lactobacillus plantarum*, transcriptomics, antioxidant response

## Abstract

This study was aimed to gain new insights into the molecular mechanisms used by *Lactobacillus plantarum* WCFS1 to respond to hydroxytyrosol (HXT), one of the main and health-relevant plant phenolics present in olive oil. To this goal, whole genome transcriptomic profiling was used to better understand the contribution of differential gene expression in the adaptation to HXT by this microorganism. The transcriptomic profile reveals an HXT-triggered antioxidant response involving genes from the ROS (reactive oxygen species) resistome of *L. plantarum*, genes coding for H_2_S-producing enzymes and genes involved in the response to thiol-specific oxidative stress. The expression of a set of genes involved in cell wall biogenesis was also upregulated, indicating that this subcellular compartment was a target of HXT. The expression of several MFS (major facilitator superfamily) efflux systems and ABC-transporters was differentially affected by HXT, probably to control its transport across the membrane. *L. plantarum* transcriptionally reprogrammed nitrogen metabolism and involved the stringent response (SR) to adapt to HXT, as indicated by the reduced expression of genes involved in cell proliferation or related to the metabolism of (p)ppGpp, the molecule that triggers the SR. Our data have identified, at genome scale, the antimicrobial mechanisms of HXT action as well as molecular mechanisms that potentially enable *L. plantarum* to cope with the effects of this phenolic compound.

## 1. Introduction

Hydroxytyrosol (3,4-dihydroxyphenylethanol, HXT) is one of the most abundant phenolic compounds in olive oils [1]. The biological effects of HXT have been investigated considerably as they seem to account for some of the health benefits associated with the consumption of olive oil.

These potential benefits have been mainly associated with the antioxidant capacity of this phenolic compound [2,3]. However, a known biological effect of HXT involves the antimicrobial activity displayed against a wide range of microorganisms, including bacteria [2]. The antimicrobial effects of HXT suggest that this compound may generate reactive oxygen species (ROS) given that antimicrobials usually share the production of these molecular species as mechanism to kill bacteria [4]. Supporting this hypothesis, other plant phenolic compounds that display antimicrobial capacity, including tannic acid [5,6] or p-coumaric acid [7], also generate ROS and have been observed to elicit antioxidant responses in *Lactobacillus plantarum*, a bacterium species associated with the olive and olive food products.

On account of its antimicrobial properties, HXT could influence host health status by shaping gut microbial communities given the recognized role of gut microbiota in host health. Lactobacilli are successfully adapted to olive and mammalian gut habitats and display higher tolerance to phenolic compounds than other bacterial groups [8]. Increasing the insights into the molecular mechanisms by which HXT exert selective pressures on lactobacilli could advance the understanding of transformation changes induced by this olive phenolic compound in the gut or olive microbiota. For example, HXT could influence the structure of the microbiota of the small intestine, which is the site where fats, including olive oil, are absorbed and where *Lactobacillus* species constitute a major bacterial population [9]. Thus, shifts triggered by HXT towards gut microbial groups such as Lactobacilli, which are regarded as safe, may contribute to improve the host health status.

*Lactobacillus plantarum* is a suitable microorganism to characterize the molecular responses to HXT. This bacterium is present in the olive and olive oil microbiota, is also a member of the olive orchard microbiome and colonizes the gastrointestinal (GI)-tract from animals. In addition, *L. plantarum* can be only inhibited at high HXT concentrations [10]. Increasing the insights into the effects of HXT on gene transcription may help to elucidate in more detail the molecular mechanisms underlying the effects of HXT at genome scale in whole biological systems, such as *L. plantarum*, which are currently fragmentary [2]. These insights can provide a knowledge base for using HXT to, for instance, improve the antioxidant potential of lactobacilli, better manage the gut or olive microbiomes or refine the use of olive oil as a vehicle for probiotic microorganisms. From a fundamental viewpoint this knowledge may help to better understand the antimicrobial effects of HXT and the mechanisms of bacterial adaptation to this compound. In the same vein, this knowledge would expand the current catalog of transcriptional datasets that report the gene and gene-circuits used by this microorganism to adapt to other plant phenolic compounds [5,6,7,11,12,13,14] as well as to plant niches [15,16] including olive oil [10]. In view of the above mentioned, the objective of this work is to study the contribution of differential gene expression in the adaptation to HXT, the main olive oil phenolic compound, as characterized by whole genome profiling in *L. plantarum* WCFS1.

## 2. Material and Methods

### 2.1. Bacterial Strain, Culture Conditions

*Lactobacillus plantarum* WCFS1 was kindly provided by Dr. Michiel Kleerebezem (NIZO Food Research, Ede, The Netherlands) and grown in Man-Rogosa-Sharpe (MRS) broth (Difco Laboratories, Madrid, Spain) [17] at 30 °C without shaking.

### 2.2. RNA Extraction

Twelve paired independent *L. plantarum* WCFS1 batch cultures (50 mL each) were grown in MRS to an OD_600_ ≈ 0.8–0.9. Then, a culture of each pair (twelve cultures) was centrifuged at 9000× *g* for 5 min at 20 °C and cells suspended in MRS containing HXT medium (10 mM final concentration) (Seproxbiotech, Madrid, Spain), which was freshly prepared prior to use. After 10 min of exposure, the HXT-induced cells and their respective controls were centrifuged at 4 °C. The pellet was mixed with 2 mL of quenching buffer (60% methanol, 66.7 mM HEPES, pH 6.5, −40 °C). Following quenching, the cells were centrifuged at 9000× *g* for 10 min at −10 °C and suspended in an extraction mixture (500 µL 1:4 chloroform-acid phenol, 30 µL of 10% SDS, 30 µL Na-acetate 3M pH 5.2, 400 µL Tris-EDTA buffer [10 mM Tris(hydroxymethyl) amino methane, 1 mM EDTA] pH 7.4, 15 mg of polyvinylpolypyrrolidone, and 500 mg of glass beads (φ, 75–150 m). The cells were broken in a FastPrep^TM^ Fp120 (Thermo Savant, Holbrook, NY, USA) by applying three cycles at 5000 rpm for 40 seconds with chilling in dry ice for 1 min between cycles. The suspension was then centrifuged at 4 °C at 10,000× *g* for 2 min. After two extractions with 500 L of chloroform, the supernatant containing the RNA was immediately frozen in liquid nitrogen and stored at −80 °C [18]. NanoDrop ND1000 instrument (Thermo Scientific, Madrid, Spain) was used for RNA quantification. The A_260_/A_280_ and A_260_/A_230_ ratios were measured to check RNA purity. Integrity and quality of RNA samples were determined by electrophoresis on agarose gels. Two treatments with DNase I (Ambion, Inc., Carlsbad, CA, USA) were applied and the absence of genomic DNA was confirmed by PCR [18].

### 2.3. Microarray: cDNA Synthesis, Purification and Hybridization

Before first-strand cDNA synthesis, RNA integrity was evaluated using the Agilent 2100 Bioanalyzer (Agilent Technologies, Inc., Santa Clara, CA, USA). Fluorescently labelled cDNA was obtained by using the SuperScript Indirect cDNA Labeling System (Invitrogen Carlsbad, CA, USA). After, the Cy3 and HyPer5 fluorescent dyes (Amersham Pharmacia Biotech, Buckinghamshire, UK) were coupled to the aminoallyl-modified first-strand cDNA, and purification of probes was carried out with the CyScribe GFX Purification Kit (GE Healthcare, Madrid, Spain). Labeling efficiency was assessed using a NanoDrop ND1000 spectrophotometer. Preparation of probes and hybridization at 65 °C during 17 h was performed as described on the Two-Color Microarray Based Gene Expression Analysis Manual (Quick Amp Labeling, Agilent Technologies, Madrid, Spain) with Tecan HS Pro Hybridization (V. 5.7/Agilent Technologies, Madrid, Spain). Slide *L. plantarum* WCFS1 8x15K microarray GE Agilent G2509F Oligo Microarrays (No. 026636) (Agilent Technologies, Madrid, Spain) was custom designed and contains 60-mer probes that were taken at the gene expression omnibus database (GEO Accession No.GPL5874). The oligo-microarray contained an average of three probes per transcript.

Real-time quantitative RT-PCR assays (RT-qPCR). Real-time RT-qPCR was used to validate the microarray data. Amplification was carried out using a 7500 Fast System (Applied Biosystems, Inc., Foster City, CA, USA). RNA was reverse transcribed using High Capacity cDNA Reverse Transcription Kits (Applied Biosystems). The specific primers used for the RT-qPCR assays are listed in the Supplementary Table S1. The SYBR Green method was used, and each assay was performed in triplicate using SYBR Green real-time PCR Master Mix (Applied Biosystems). Amplification was initiated at 95 °C for 10 min, followed by 40 cycles of 95 °C for 15 s and 60 °C for 1 min. Control PCRs were included to confirm the absence of primer dimer formation (no-template control), and to verify that there was no DNA contamination (without RT enzyme negative control). All real-time PCR assays amplified a single product as determined by melting curve analysis and by electrophoresis. A standard curve was plotted with cycle threshold (Ct) values obtained from amplification of known quantities of cDNAs and used to determine the efficiency (E) as E = 10^−1/slope^. The expression levels of target genes were normalized. The Bestkeeper analysis [19] as applied and the geometric mean of the most stably expressed housekeeping genes (16S rRNA, *gapB, dnaG*, and *gyrA*) was used as a normalization factor. The expression ratios measured by microarrays and by RT-qPCR assay were plotted, and the linear correlation coefficient was calculated (y = 1.3163x-1.0264; R^2^ = 0.94).

### 2.4. Data Analysis

Images were captured with a GenePix 4000B (Axon, Molecular Devices, San Jose, CA, USA) and spots quantified using GenPix software (Axon, Molecular Devices, San Jose, CA, USA). Background correction and normalization of expression data were performed using the methods normexp and loess in LIMMA, respectively [20]. The expected False Discovery Rate (FDR) was controlled to be less than 5%. Genes were considered as differentially expressed (DE) when nominal *p*-values were < 0.05 and had a fold change (FC) equal or higher than ± 1.5 FC was calculated as the average of the fold change between significantly regulated probes. Hybridizations and statistical analysis were performed by the Genomics Facility at the National Center for Biotechnology, CSIC, Madrid, Spain.

Finally, genes showing a 1.5-fold or greater FC (*p* < 0.05) were used to conduct a functional enrichment analysis according to their GO and KEGG’s pathway terms by using DAVID [21] (https://david.ncifcrf.gov). Terms with *p*-values less than 0.1 were considered as significant.

### 2.5. Microarray Data Accession Number

The microarray data provided in this study have been deposited in NCBI Gene Expression Omnibus [22] genomics data repository and are accessible through GEO Series accession number GSE90714.

## 3. Results and Discussion

### 3.1. Global Analysis of the Lactobacillus Plantarum wcfs1 Transcriptomic Response to Hydroxytyrosol

**Figure 1:**
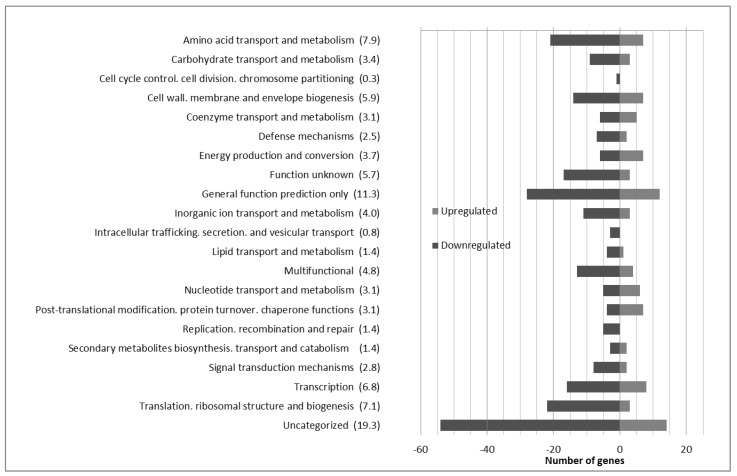
Hydroxytyrosol-regulated genes from *Lactobacillus plantarum* WCFS1 classified by functional categories. The number of upregulated and downregulated genes (percentage in parentheses) in the presence of 10 mM hydroxytyrosol is shown for each category.

To bring new knowledge into the response of *L. plantarum* WCFS1 to HXT, a global transcriptomic analysis was carried out using DNA microarrays. The analysis showed 314 DE genes after 10 min exposure to HXT, as compared to control conditions (*t* = 0). From these genes 90 were upregulated and 224 downregulated. The DE genes can be checked in Supplementary Table S2. These genes were functionally classified according to their Clusters Orthologous Groups (COGs) categories (Figure 1).

**Figure 2:**
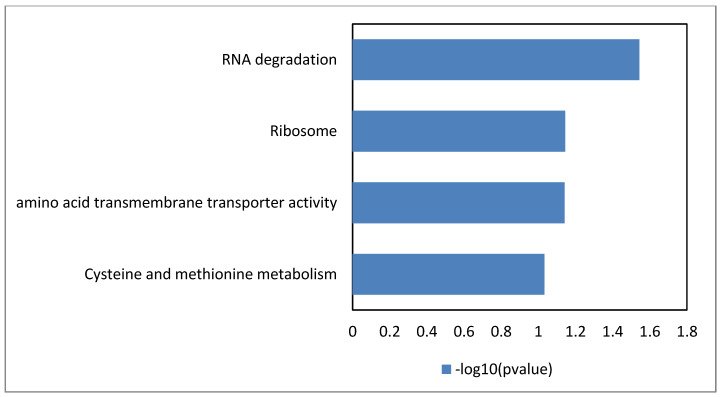
DAVID analysis of functional gene ontology (GO) categories and KEGG pathways related to the *Lactobacillus plantarum* genes differentially expressed in 10 mM hydroxytyrosol. Fold enrichment (bars) parameter measures the magnitude of enrichment, both for up and down-regulated gene groups.

The enriched GO-DIRECT categories and KEGG pathways resulting from the Gene Ontology (GO) analysis carried out with the DAVID tool are shown in Figure 2. This analysis reveals cysteine and methionine metabolism, amino acid transmembrane transporter activity, as well as ribosome and RNA degradation as the most significantly enriched biological processes.

### 3.2. Antioxidant Responses Triggered by Hydroxytyrosol

**Scheme 1:**
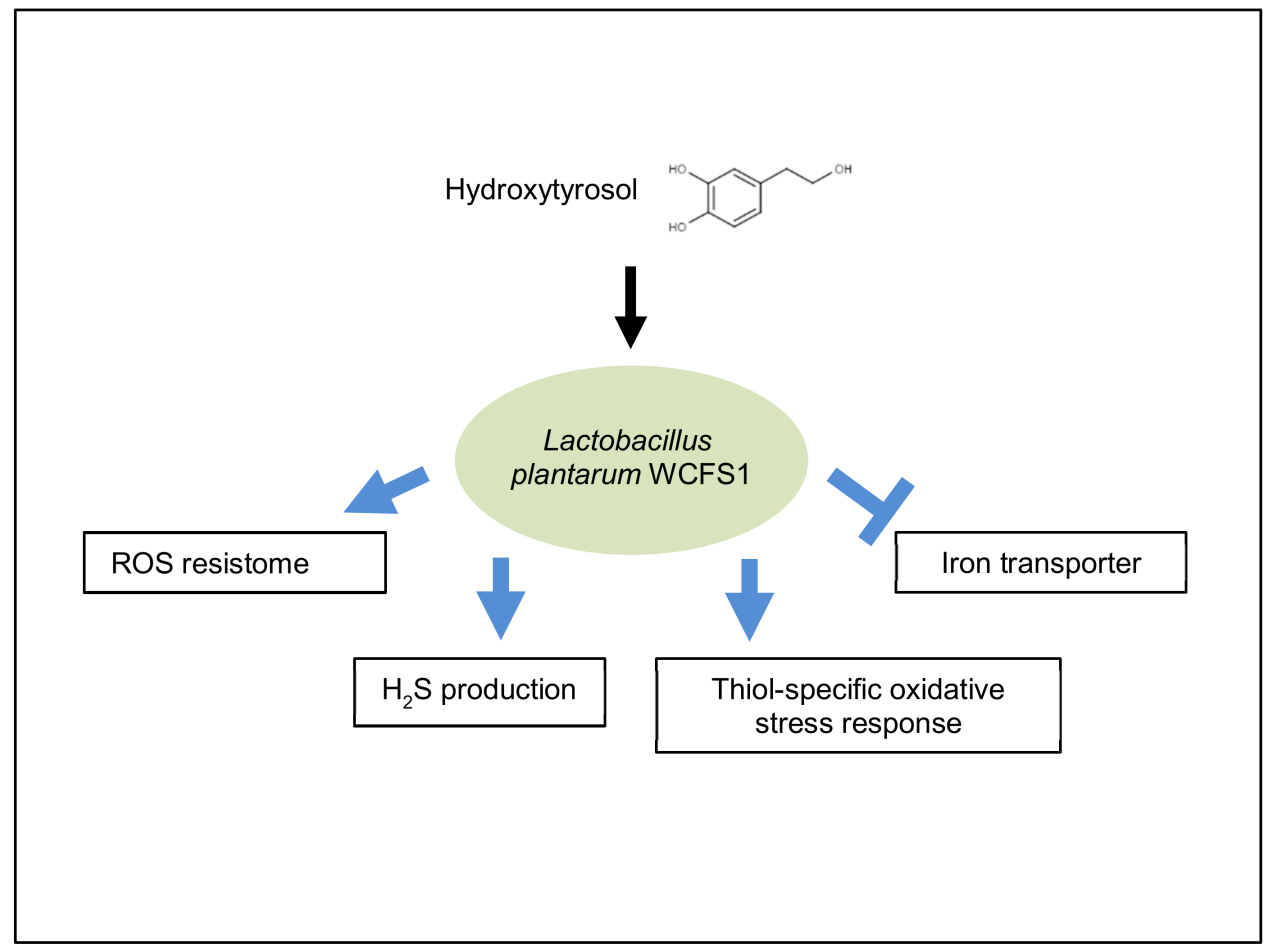
Schematic view of molecular components involved in *the Lactobacillus plantarum* antioxidant response to hydroxytyrosol. Arrows represent upregulated components; perpendicular bars, downregulated components. ROS, reactive oxygen species.

The presence of HXT elicited antioxidant mechanisms mediated by genes coding for enzymes known to counter oxidative stress (Scheme 1).

Some of these enzymes are encompassed within the ROS resistome of lactic acid bacteria [23], including *kat* or *lp_3578* (catalase), *lp_3587* or *pox4* (pyruvate oxidase) and *lp_2544* or *npr2* (NADH-peroxidase), which are dedicated to inactivate or prevent the formation of toxic oxygen radicals. This profile was accompanied by downregulation of *lp_2992* or *mntH3*, a membrane protein-encoding gene previously linked in *L. plantarum* WCFS1 to the transport of iron [24], which is a metal precursor to ROS-induced damage via Fenton chemistry.

In addition, the response of *L. plantarum* WCFS1 to HXT revealed the induction of genes coding for enzymes involved in counteracting thiol-specific oxidative stress in this microorganism [25], including *msrA3* (methionine sulfoxide reductase), *gshR4* (glutathione reductase) or *lp_0858* (regulator of disulfide bond formation). Furthermore, the cystathionine-β-synthase (CBS) (*lp_0255*) and cystathionine-γ-lyase (CSE) (*lp_0256*) encoding genes, which have been recently shown as the most important H_2_S-generating enzymes in many bacteria [26], were also up-regulated. While the role of H_2_S is disputed [27] it has been proposed that this gas, when produced endogenously by CBS and CSE, partially neutralize the effectiveness of the oxidative stress that, regardless of the diversity of their action, antibiotics induce [4,28]. Notably, the glutathione antioxidant network [29] and the expression of antioxidant enzymes such as catalase [29,30] have been also elicited by HXT in other biological systems, which support the expression profiles found in this study.

Overall, the observed gene expression patterns, including the induction of genes encompassed within the ROS resistome of lactic acid bacteria, the down-regulation of an iron transporter, the induction of genes coding for enzymes counteracting thiol-specific oxidative stress and the up-regulation of H_2_S-generating enzymes suggest that HXT acts as an oxidative stressor to *L. plantarum* WCFS1. These effects are in line with the responses to counteract the oxidative stress generated by other phenolic compounds with antimicrobial activities in *L. plantarum* WCFS1, such as tannic acid [5] or p-coumaric acid [7].

### 3.3. RNA Degradation

Genes encompassed within the GO term “RNA degradation” were significantly enriched in the DAVID analysis (Figure 2). These genes, which encode proteins that are components of the RNA degradosome, were differently regulated by HXT. Thus, *lp_0792* or *enoA1* (enolase), *lp_2122* (RNase J) and *lp_2278* or *rhe3* (RNA helicase highly homologous to the CshB RNA helicase from *Bacillus subtilis*) were downregulated whereas *lp_1898* or *pfk* (6-phosphofructokinase), *lp_0842* or *ppk* (polyphosphate kinase) and *lp_0728* or *groEL* (chaperonin GroEL), were upregulated. The RNA degradosome is believed to degrade RNA with high efficiency [31]. This protein complex has been suggested as a mechanism potentially degrading oxidized RNA [32], but it is also involved in maintaining the typical half-lives of RNA transcripts so that relatively low amounts of oxidized RNA molecules should be degraded to maintain a functional RNA repertoire. In view of this, downregulation of *lp_2122* (RNase J) and *lp_2278* or *rhe3* (RNA helicase) may have helped to adjust transcript abundance to adapt *L. plantarum* to HXT stress whereas upregulation of genes coding for accessory proteins of the degradosome (*pfk*,, *groEL*) may have stabilized the protein complex and remove poly-P (*ppk*), which is inhibitory for RNA degradation.

### 3.4. Elements Typically Involved in the Stringent Response Are Responsive to HXT

The transcriptomic analysis revealed the involvement of the stringent response (SR), a bacterial process that can be triggered by a broad variety of stress conditions [33], in the adaptation of *L. plantarum* to HXT. Among the transcriptional alterations triggered by HXT that concern the SR were those affecting the expression of genes involved in cell proliferation. Thus, genes encompassed within the GO term “ribosome” were significantly enriched in the DAVID analysis (Figure 2). Among these, a set of nine rRNA genes (*lp_0011* [rpsR], *lp_1063* [rplQ], *lp_1078* [rpsI], *lp_2125* [rpsO], *lp_2126* [rpsT], *lp_0543* [rpsS1], *lp_1594* [rpl27], *lp_1640* [rplS], *lp_1517* [rplT],) and a ribosomal protein–serine acetyltransferase (*lp_2840*) were all downregulated. In addition, other genes coding for translation related functions including *lp_2378* or *prfA* (peptide chain release factor), *lp_0963* or *rluE* (ribosomal large subunit pseudouridine syntethase), *lp_2039* or *rbfA* (ribosome binding factor), *lp_2277* or *alaRS* (alanyl-tRNA syntethase and *lp_1027* or *fusA2* (elongation factor), were downregulated (Supplementary Table S2). Also, other genes related to cell proliferation that code for factors playing roles in DNA (*lp_0787* or *rpoN*; *lp_0811* (DNA-directed DNA polymerase III subunit epsilon; *lp_2454* (DNA adenine methylase)) or RNA replication (*lp_1613* or *rpoZ*), were downregulated. In the same vein, genes coding for cell division (*lp_2201* or *ftsL*) or cell shape determination (*lp_2317* or *mreD*) were downregulated in the presence of HXT. Furthermore, genes that are part of the SR with known roles in the adaptation to stress-related processes such as those coding for the alkaline shock proteins *asp1*, and *asp2* or the molecular chaperones *groEL* and *groES*, were up-regulated.

Other transcriptional alterations elicited by HXT owing to the SR concerned the metabolism of (p)ppGpp, the nucleotide-based secondary messenger that acts as alarmone to trigger the SR. The (p)ppGpp is interlinked and modulates intracellular purine concentrations since GTP mediates in the biosynthesis of this alarmone. HXT regulated the expression of several genes involved in purine metabolism (Supplementary Table S2). The expression of genes involved in GTP biosynthetic pathways (purine biosynthesis) such as *lp_0848* (purine transporter), *lp_2710* (xanthine permease) and *lp_3269* or *purB* (adenylosuccinate lyase, AMP synthesis) were downregulated whereas *lp_2727* or *purC* (phosphoribosyl aminoimidazole succinocarboxamide synthase, IMP synthesis) was upregulated. Genes involved in GTP-consuming pathways such as *lp_1869* or *dfrA* (dihydrofolate reductase, folate biosynthesis) and *lp_1436* (riboflavin synthase) were upregulated upon HXT which may also interfere with the synthesis of (p)ppGpp via intracellular GTP consumption. In addition, HXT modulated the expression of genes coding for enzymes that are directly involved in the interconversion of (p)ppGpp to ppGpp such as small translational GTPases (*lp_1881* or *engA*; *lp_1687* or *rsgA2*). Also, a GTP pyrophosphokinase (*lp_0293*) with high homology to other (p)ppGpp synthetases (*Staphylococcus aureus relP*), was upregulated. Concomitant with this profile *lp_2114* coding for an NTP pyrophosphohydrolase with high homology with *mazG* (an enzyme with GTP hydrolytic activity [34]) and a phosphohydrolase encoding gene (*lp_1985*) located in the vicinity of *relA* (*lp_1988*), were downregulated. Genes coding for the stringent response factors (enzymes) exopolyphosphatase (*lp_3587* or *ppx3*) and pyrophosphokinase (*lp_3578* or *ppk*) which control the intracellular levels of inorganic phosphate (poly-P), a key regulatory molecule in the stringent response [35], were upregulated. The regulation of genes involved in the SR, a highly conserved stress defense mechanism, indicates that HXT causes stress to *L. plantarum* WCFS1. Interestingly the SR was also triggered in *L. plantarum* WCFS1 in presence of olive oil. Albeit HXT is the main phenolic compound in extra virgin olive oil (EVOO) [10], it cannot be ruled out the likelihood that other components present in this food matrix, including other phenolic compounds or fatty acids, may also contribute to elicit the SR. Indeed, although the processes owing to the SR affected by HXT and EVOO were the same, the genes involved such as rRNA genes, genes coding for cell division, genes involved in pathways that determine the GTP/ATP ratio and intracellular phosphate pools, on which depends the accumulation of the alarmone (p) ppGpp, differed in both conditions. This could be expected as sensing systems, enzymesm and pathways involved in the SR can differ depending on the stress signals that trigger this process [36].

### 3.5. Cell Envelope Modifications in Response to HXT Stress

*L. plantarum* adjusted the expression of a set of genes coding for proteins involved in the biogenesis of the cell wall, including several glucosyltransferases that were differently regulated by HXT. Genes *lp_2844* or *tagE6* (poly (glycerol-phosphate) α-glucosyltransferase) and *lp_1818* or *tagB1* were upregulated whereas *lp_1248* or *tagD2*, *lp_1372* or *gtcA1* (a protein involved in teichoic acid glycosylation), and *lp_2716* or *ica3* as well as *lp_2783* (both glycosyltransferases), were downregulated. This profile could indicate modification of the wall teichoic acid (WTA) by glycosyl substitution in response to HXT.

Genes coding for other proteins involved in cell wall biogenesis were also differently expressed upon HXT. Gene *lp_1713* or *lysA* (diaminopimelate (DAP) decarboxylase, which catalyzes the final step in the meso-DAP/lysine biosynthetic pathway) was downregulated, which can avoid DAP dissipation towards lysine production and provide more meso-DAP to be incorporated as constituent of the peptidoglycan (PG) layer of *L. plantarum* cell walls. Interestingly *L. plantarum* uses a similar strategy to respond to the damage incurred by tannic acid in the cell wall, i.e., overproducing DapF protein (DAP epimerase) which epimerizes L, L-DAP into meso-DAP to be incorporated into the PG [5]. The strategy of increasing meso-DAP for PG biosynthesis in response to HXT may similarly reflect damage in the cell wall caused by this phenolic compound. Another enzyme that participates in PG biosynthesis of *L. plantarum*, *lp_2199* or *mraY* (phospho-N-acetylmuramoyl-pentapeptide-transferase) was downregulated. MraY is an essential enzyme that catalyzes the first membrane step of bacterial cell wall PG synthesis and is a promising target for the search of new antibacterial compounds [37]. Hence the observed profile identifies MraY as a target for the antibacterial action of HXT.

A set of different genes coding for enzymes that can hydrolyze specific bonds in PG was downregulated, probably as strategy to maintain cell wall integrity. Among these, three code for enzymes of the peptidoglycan hydrolase complement (PGH) including two extracellular lytic transglycosylases (*lp_3015*; *lp_0302*) and the N-acetylmuramoyl –L-alanine amidase (*lp_1982* or *lytH*). Also, a diaminopimelate muropeptidase (*lp_2162*) as well as a gene couple (*lp_3254* or *lgrA*, *lp_3255* or *lgrB*) which code for a murein hydrolase export protein and a murein hydrolase regulator, were also downregulated.

Amassing of compatible solutes in the extracellular space seems to be a further strategy to avoid damage in the cell envelope caused by HXT, judging by the downregulation of several transporters for these substances. These included the *lp_3324* (glycine betaine/carnitine/choline transporter), *lp_1722* (GABA transporter) and *lp_0265* (trehalose PTS transport system) genes, which is coincident with the response of *L. plantarum* to p-coumaric acid [7], a phenolic compound that turns leaky the membrane of several bacteria. It was also observed an increased expression of three subunits of the H^+^-ATPase (*lp_2365* or *atpG*, *lp_2367* or *atpH* and *lp_2365* or *atpF*). The increased expression of F_0_F_1_-ATPase subunits may be required to compensate for a potential proton motive force dissipation caused by HXT damage on membrane integrity, as it has been speculated to occur upon exposure to bile acids [38], albeit this hypothesis needs to be experimentally supported. Interestingly *atpF* subunit was downregulated in the presence of EVOO [10]. In this case, F_0_F_1_-ATPase subunit downregulation is modulated by fatty acids and is probably linked to diminished respiration metabolism triggered by fatty acid pathway disfunction.

The observed response, including the downregulation of enzymes that hydrolyze specific bonds in the PG, differential regulation of genes involved in PG biosynthesis, WTA modification, augmented expression of F_0_F_1_-ATPase, and amassing of compatible solutes in the extracellular space, indicates that HXT injures the cell envelope of *L. plantarum*, a subcellular compartment impacted by different types of stressors [27]. This expression profile is in line with the transcriptomic and proteomic responses of *L. plantarum* to other phenolic compounds with antimicrobial activity that injure the cell envelope, such as tannic acid [5,6] or p-coumaric acid [7], which also lead to differential expression of the same or similar functions involved in the protection and biogenesis of the cell envelope.

### 3.6. Energy Metabolism

The gene coding for a mannitol-specific PTS-system component (*lp_0230* or *pts2CB*) and the gene *lp_1898* or *pfkA* (6-phosphofructokinase) were up-regulated. This expression profile could be expected from a bacterium that is closely associated to the olive where mannitol and fructose are main components of olive fruits [39]. *L. plantarum* downregulated two malate transport proteins (*lp_0594* and *lp_2829*) and increased the expression of a malolactic regulator (*lp_1116*). The downregulation of malate transport probably indicates a reduced contribution of the known functionality of malate transport and metabolism to chemiosmotic mechanisms of energy generation and pH homeostasis [40].

### 3.7. Nitrogen Metabolism

The transcriptomic response of *L. plantarum* to HXT involved the differential expression of genes encompassed by the GlnR regulon. This response included the downregulation of genes involved in the influx and production of glutamine and ammonium, i.e., *lp_1581* or *glnA* (glutamine synthetase), *lp_0822* (glutamine-fructose-6-P transaminase), genes coding for two Gln ABC transporters (*lp_0802* and *lp_0803*) (*lp_2110* and *lp_2111*), *lp_2830* (aspartate ammonia lyase which produces fumarate from Asp with the production of ammonium), *lp_0956* or *asnC* (Asn-tRNA ligase) and *lp_0349* or *amtB* (NH_4_^+^ transport protein). In addition, *lp_2738*, coding for L-asparaginase was upregulated. This response prevents the influx and simultaneously limits the intracellular production of ammonia and glutamine. It allows for a tight control and impairment of efflux of ammonia levels which in turn are required to ensure sufficient biosynthetic nitrogen assimilation for growth. The observed profile regarding nitrogen metabolism is very well conserved among the responses of *L. plantarum* WCFS1 to other phenolic compounds, including gallic acid [11], resveratrol [13], oleuropein [14], and p-coumaric acid [7].

### 3.8. Regulation of ABC-Transporters

The response to HXT showed a reduced transcription of two ABC-transporters (*lp_2739*-*lp_2740* and *lp_2743*- *lp_2744*) that were downregulated in response to other phenolics such as GA [11] and p- coumaric acid [7], resveratrol [12,16] or oleuropein [13,17]. The gene pair *lp_2739*-*lp_2740* codes for an ABC transporter homologous to the BceAB transporter of *Strepcoccus pneumoniae* [41] or its ortholog YsaBC of *Lactococcus lactis*. This BceAB-like module counteract the activity of bacitracin in *B. subtilis*, an antibiotic that blocks the lipid II cycle and is associated to cell envelope stress response in LAB [28]. This BceAB system has not been studied in *L. plantarum* and thus substrates, mode and direction of substrates transport are unknown [42]. The genes *lp_2743*-*lp_2744* constitute an ABC-transport system homologous to that encompassed by the YtrA operon from *Bacillus subtilis* [43] which often responds to cell wall antibiotics. To judge by these expression profiles, *L. plantarum* WCFS1 could downregulate these transport systems to control the traffic of HXT and other phenolic compounds across the membrane. In addition, *lp_3040*, a gene putatively coding for a multidrug efflux pump, was upregulated. Like other multidrug resistance systems [44], this transporter is probably dedicated to remove various substances from the cytoplasm, among which could be HXT.

## 4. Conclusions

By using a transcriptomic approach, new insights have been found regarding the mechanistic interactions between *Lactobacillus plantarum* and HXT, the main olive oil phenolic compound. According to the datasets, this microorganism elicits antioxidant mechanisms known to counter oxidative damage, including the induction of genes known to be part of the ROS resistome, genes involved in the response of this microorganism to thiol-specific oxidative stress, genes coding for H_2_S-generating enzymes and a response to decrease the load of copper, a metal promoting oxidative damage. This response supports the hypothesis that, similarly to other antimicrobial compounds, the antimicrobial effects of HXT involve ROS overproduction as a mechanism to kill bacteria. Pre-conditioning schemes using HXT as stressor may increase the antioxidant capacity of beneficial strains such as *L. plantarum* WCFS1. To judge by the modulation of a set of genes involved in the protection and biogenesis of the cell envelope, this subcellular compartment is also a major target for HXT. Other transcriptomic responses to HXT, such as the stringent response or changes in the expression of MFS efflux systems and ABC-transporters, also reflect the antimicrobial stress mediated by HXT. The results of this study have identified antimicrobial mechanisms of HXT action and adaptation mechanisms of *L. plantarum* to this olive phenolic compound. This improved knowledge may be useful to better understand the transformation changes in bacterial communities such as the gut or plant microbiomes and potentially be used to select/improve beneficial microorganisms like *L. plantarum* that better adapt to HXT-associated stress.

## Supplementary Materials

The following are available online at https://www.mdpi.com/2076-3921/9/5/442/s1, Table S1: Oligonucleotides used for qRT-PCR in this study designed with Primer Express 3.0 software. Table S2: *Lactobacillus plantarum* WCFS1 genes with differential expression in presence of 10 mM hydroxytyrosol.
