# Supplementary material for: Transcriptomic Evidence of Molecular Mechanisms Underlying the Response of Lactobacillus plantarum WCFS1 to Hydroxytyrosol
Source: Antioxidants (Basel). 2020 May 20;9(5):442. doi: 10.3390/antiox9050442 (PMC7278804; doi:10.3390/antiox9050442)
Supplement: Supplementary file 1 [file antioxidants-09-00442-s001.pdf]

antioxidants

MDPI

Article

Transcriptomic evidence of molecular mechanisms underlying the response of *Lactobacillus plantarum* WCFS1 to hydroxytyrosol

Inés Reverón[1], Laura Plaza-Vinuesa[1], Laura Santamaría[1], Juan Carlos Oliveros[2], Blanca de las Rivas[1], Rosario Muñoz[1] and Félix López de Felipe[1*]

[1] Laboratorio de Biotecnología Bacteriana. Instituto de Ciencia y Tecnología de los Alimentos y Nutrición (ICTAN-CSIC), 28040 Madrid. Spain

[2] National Center for Biotechnology (CNB-CSIC), 28049 Madrid, Spain

* Correspondence: Felix Lopez de Felipe, Laboratory of Bacterial Biotechnology, ICTAN-CSIC, Ciudad Universitaria s/n, Madrid 28040, Spain, Fax: +34 91 549 36 27, E-mail: fxlopez@ictan.csic.es

Received: date; Accepted: date; Published: date

antioxidants MDPI

Table 1. Oligonucleotides used for qRT-PCR in this study designed with Primer Express 3.0 software.

Locus Tag[a]	Locus	Description	Primer sequence (5´ ⟶ 3´)	Expression ratios[d]	
				Microarray[e]	RT-qPCR[f]
lp_2108	*cps4A*	polysaccharide biosynthesis protein, chain length regulator	F[b]: TTGGGCGGGACGATTG<br>R[c]: GCTTGATATTCGGGCTGAATG	1.05	0.17
lp_0255	*metC1*	cystathionine beta-lyase / cystathionine gamma-lyase	F: TTGGCCCCGCAAGAAA<br>R: ATGCGCAATGCCAGAGTCT	1.03	0.55
lp_0843	*ppx3*	exopolyphosphatase	F: GCCGTTTGCCACCCTCTT<br>R: GTTCCTCCACGCCAGCTTT	1.1	0.32
lp_0927	_	membrane protein	F: CCAACAGGCGGCAACCT<br>R: TTCGACCGTCAATCCCAATT	1.18	0.08
lp_0291	_	glycolate dehydrogenase, subunit GlcD,FAD-binding	F: CCGGACCCTGGTTCTAAGC<br>R: GGCGTTTGTCGCCACATTAC	1.04	0.78
lp_3184	_	branched-chain amino acid transport protein	F: TGGCTTTCATACCACGCTTTT<br>R: TTGGGATCTTCCGCTTCGT	-1.76	-2.54
lp_2525	_	ABC transporter ATP-binding protein	F: GCAGCGCAACGTTCGAA<br>R: ATGCCGGCTGGAATATCG	-1.71	-3.23
lp_3254	*lrgA*	murein hydrolase export protein	F: AGGTGGCGGGTGTTAGCA<br>R: TCCCTGATGGAATGAAAATCAAG	-1.94	-3.92
lp_0349	*amtB*	ammonium transport protein	F: GGCATGGTCGGCAGTATCAT<br>R: TTGTGCGATTAACGGCTTTG	-1.77	-2.9
lp_2740	_	ABC transporter permease	F: CAACCTAACCGGCCACAACT<br>R: GACACCGACGACAATTTCGA	-1.94	-4.47
**Housekeeping and others internal control genes**					
lp_2057	*ldhD*	D-lactate dehydrogenase	F: AACCGCGACAATGTTTTGATT<br>R: TTGTGAACGGCAGTTTCAGTGT	-1.47	-2.96
lp_0789	*gapB*	glyceraldehyde 3-phosphate dehydrogenase	F: CTGGTGCTGCTAAGGCTCTTG<br>R: TGTGCATGGCCTTGTAATTTACC	-0.25	-1.35
lp_1963	*dnaG*	DNA primase DnaG	F: TCCGGAAGCAGTCGTCAAG<br>R: TCGCCGGCAAGTCAATGT	-0.13	-1.19
lp_0007	*gyrA*	DNA gyrase, A subunit	F: CCCGACAGCAACGTCTTCA<br>R: GGCAGCTGGCGTTTGTTT	-0.05	-1.09

antioxidants MDPI

lp_1962	*rpoD*	RNA polymerase sigma factor RpoD	F: CGGATCCGCCAAATCG<br>R: CGTGATGGGTGGCGTAACTT	0.03	0.98
lp_0006	*gyrB*	DNA gyrase, B subunit	F: CCCGGGTCGCTGCTAAG<br>R: TTTCCAAGCCACTCTTTTTTCG	0.27	-0.67
lp_2301	*recA*	recombinase A	F: CGGCGGGCAGAACAGAT<br>R: TTTCCAAGCCACTCTTTTTTCG	-1.1	-2.47
lp_1021	*rpoB*	DNA-directed RNA polymerase subunit beta	F: GGGTGTGCCTTCTCGTATGAA<br>R: CAGCCATCCCCAAATGCA	0.02	-1.0
lp_rRNA01	16srRNA	16S ribosomal rRNA	F: GGGTAATCGGCCACATTGG<br>R: CTGCTGCCTCCCGTAGGA	0.26	-0.68

[a] Designated gene number for the annotated *L. plantarum* WCFS1 genome. [b] Forward. [c] Reverse . [d] Genes ratios >1.5 fold changes (either increase or decrease) were statistically significant ($p < 0.05$). [e] log2ratio(M), where ratio(M) = fold change. [f] log2 ratios of average fold change.

Table 2. *Lactobacillus plantarum* WCFS1 genes with differential expression in presence of 10 mM hydroxytyrosol.

Gene ID	Locus	Description	COGs	Fold Change[a,b]	Subcellular Localization Prediction[c]	Pathway Prediction[c]
lp_0011	*rpsR*	30S ribosomal protein S18	J: Translation, ribosomal structure and biogenesis	-2,15	Intracellular	
lp_0030	_	pyridoxamine 5'-phosphate oxidase family protein, FMN-binding	_	-1,63	Intracellular	
lp_0032	_	hypothetical protein	_	-1,9	Intracellular	
lp_0039	_	regulator of two-component system, YycI family	S: Function unknown	-1,93	N-terminally anchored (No CS)	Sec-(SPI)
lp_0047	*aldH*	coniferyl aldehyde dehydrogenase	C: Energy production and conversion	1,65	Intracellular	
lp_0052	_	6-pyruvoyl-tetrahydropterin synthase	H: Coenzyme transport and metabolism	-1,78	Intracellular	
lp_0069	*ptp1*	protein-tyrosine phosphatase	T: Signal transduction mechanisms	-1,7	Intracellular	
lp_0099	_	hypothetical protein	_	-1,87	Intracellular	
lp_0111		medium chain dehydrogenases/reductase (MDR)/zinc-dependent alcohol dehydrogenase	C: Energy production and conversion	-1,76	Intracellular	
lp_0119	_	NTP pyrophosphohydrolase	F: Nucleotide transport and metabolism	1,84	Intracellular	
lp_0120	_	amino acid/polyamine transport protein, APC	E: Amino acid transport and metabolism	-2,47	Multi-transmembrane	Sec-(SPI)
lp_0124	*pacL1*	cation transporting P-type ATPase	P: Inorganic ion transport and metabolism	-2,02	Multi-transmembrane	Sec-(SPI)
lp_0127	_	NADPH-dependent FMN reductase	R: General function prediction only	1,69	Intracellular	
lp_0151	_	no items found	_	-2,21	Intracellular	
lp_0159	_	short-chain dehydrogenase/oxidoreductase	I: Lipid transport and metabolism	1,70	Intracellular	
lp_0202	_	GNAT family acetyltransferase	_	-1,84	Intracellular	
lp_0215	_	ABC transporter ATP-binding protein	R: General function prediction only	-2,03	Intracellular	

lp_0230	*pts2CB*	PTS system, mannitol-specific EIICB component	G: Carbohydrate transport and metabolism	1,91	Multi-transmembrane	Sec-(SPI)
lp_0244	_	NADPH-dependent FMN reductase	R: General function prediction only	-2,23	Intracellular	
lp_0255	*metC1*	cystathionine beta-lyase / cystathionine gamma-lyase	E: Amino acid transport and metabolism	2,20	Intracellular	
lp_0256	*cysK*	cystathionine beta-synthase	E: Amino acid transport and metabolism	1,91	Intracellular	
lp_0257	*pepM*	methionyl aminopeptidase	J: Translation, ribosomal structure and biogenesis	-1,67	Intracellular	
lp_0261	_	membrane protein	_	-1,92	Multi-transmembrane	Sec-(SPI)
lp_0265	*pts5ABC*	PTS system trehalose-specific transporter subunit IIBC	G: Carbohydrate transport and metabolism	-2,77	Multi-transmembrane	Sec-(SPI)
lp_0272	_	aromatic acid carboxylyase, subunit D	_	-1,56	Intracellular	
lp_0274	_	TetR family transcriptional regulator	K: Transcription	1,55	Intracellular	
lp_0286	*pts6C*	PTS system, cellobiose-specific EIIC component	G: Carbohydrate transport and metabolism	-1,83	Multi-transmembrane	Sec-(SPI)
lp_0291	_	glycolate dehydrogenase, subunit GlcD,FAD-binding	C: Energy production and conversion	2,05	Intracellular	
lp_0292	_	hypothetical protein	_	2,11	Intracellular	
lp_0293	_	GTP pyrophosphokinase	S: Function unknown	1,5	Intracellular	
lp_0295	_	transport protein, MMPL family	R: General function prediction only	1,71	Multi-transmembrane	Sec-(SPI)
lp_0302	_	extracellular transglycosylase	_	-1,67	N-terminally anchored (No CS)	Sec-(SPI)
lp_0312	_	MarR family transcriptional regulator	K: Transcription	1,75	Intracellular	
lp_0325	_	acetoin transport repressor, GntR family	K: Transcription	-1,68	Intracellular	
lp_0327	_	acetoin ABC transporter, permease protein	_	-2,64	Multi-transmembrane	Sec-(SPI)
lp_0349	*amtB*	ammonium transport protein	P: Inorganic ion transport and metabolism	-3,40	Multi-transmembrane	Sec-(SPI)
lp_0402	_	hypothetical protein	_	-1,93	Intracellular	
lp_0444	_	hypothetical protein	_	1,85	Intracellular	
lp_0455	_	drug resistance transpor protein, major facilitator superfamily (MFS), Bcr/CflA subfamily	G: Carbohydrate transport and metabolism	-1,71	Multi-transmembrane	Sec-(SPI)

lp_0464	_	zinc/iron ABC transporter, permease protein	P: Inorganic ion transport and metabolism	-2,02	Multi-transmembrane	Sec-(SPI)
lp_0467	*glmU*	UDP-N-acetylglucosamine pyrophosphorylase	M: Cell wall/membrane/envelope biogenesis	-1,72	Intracellular	
lp_0477	_	lipoate-protein ligase A	H: Coenzyme transport and metabolism	-1,57	Intracellular	
lp_0484	*rimK*	ATP-dependent carboxylate-amine/thiol ligase	H: Coenzyme transport and metabolism	1,49	Intracellular	
lp_0509	_	hypothetical protein	_	-1,99	Intracellular	
lp_0514	*srtA*	sortase A	M: Cell wall/membrane/envelope biogenesis	1,70	N-terminally anchored (No CS)	Sec-(SPI)
lp_0535	_	hypothetical protein	T: Signal transduction mechanisms	-2,07	Intracellular	
lp_0543	_	RNA binding protein, contains ribosomal protein S1 domain	J: Translation, ribosomal structure and biogenesis	-2,57	Intracellular	
lp_0551	*dtpT*	di-/tripeptide transport protein	E: Amino acid transport and metabolism	-2,58	Multi-transmembrane	Sec-(SPI)
lp_0555	_	hypothetical protein	_	2	Intracellular	
lp_0584	_	transport protein, major facilitator superfamily (MFS)	_	-1,49	Multi-transmembrane	Sec-(SPI)
lp_0594	*mleP1*	malate transport protein	R: General function prediction only	-1,73	Multi-transmembrane	Sec-(SPI)
lp_0595	_	permease, drug/metabolite transporter (DMT) superfamily	S: Function unknown	-1,72	Multi-transmembrane	Sec-(SPI)
lp_0694	*nrdH*	glutaredoxin-like protein nrdH	O: Posttranslational modification, protein turnover, chaperones	-1,86	Intracellular	
lp_0701	_	hypothetical protein	_	-2,37	Intracellular	
lp_0726	_	membrane-bound protease, CAAX family	R: General function prediction only	-1,91	Multi-transmembrane	Sec-(SPI)
lp_0727	*groES*	GroES co-chaperonin Cpn10	O: Posttranslational modification, protein turnover, chaperones	2	Intracellular	

lp_0728	*groEL*	GroEL chaperonin Cpn60	O: Posttranslational modification, protein turnover, chaperones	1,75	Intracellular	
lp_0742	_	membrane protein	O: Posttranslational modification, protein turnover, chaperones	1,65	Multi-transmembrane	Sec-(SPI)
lp_0753	_	membrane protein	S: Function unknown	-1,61	Multi-transmembrane	Sec-(SPI)
lp_0755	*lgt*	Prolipoprotein diacylglyceryl transferase	M: Cell wall/membrane/envelope biogenesis	1,71	Multi-transmembrane	Sec-(SPI)
lp_0758	_	diguanylate cyclase/phosphodiesterase, EAL domain	T: Signal transduction mechanisms	1,71	Intracellular	
lp_0787	*rpoN*	DNA-directed RNA polymerase, sigma factor 54	K: Transcription	-1,61	Intracellular	
lp_0792	*eno*	Enolase 1 (phosphopyruvate hydratase) 2-phospho-D-glycerate hydro-lyase 1 2-phosphoglycerate dehydratase 1	G: Carbohydrate transport and metabolism	-1,83	Intracellular	
lp_0799	*smpB*	SsrA-binding protein Small protein B SSRA RNA binding protein	O: Posttranslational modification, protein turnover, chaperones	-1,96	Intracellular	
lp_0802	*glnPH1*	polar amino acid ABC transporter, substrate binding and permease protein	E: Amino acid transport and metabolism	-2,16	Multi-transmembrane	Sec-(SPI)
lp_0803	*glnQ1*	glutamine ABC transporter ATP-binding protein	E: Amino acid transport and metabolism	-2,72	Intracellular	
lp_0811	_	DNA-directed DNA polymerase III subunit epsilon	L: Replication, recombination and repair	-1,58	Intracellular	
lp_0816	_	MarR family transcriptional regulator	K: Transcription	-1,57	Intracellular	
lp_0817	_	membrane protein	S: Function unknown	-2,07	Multi-transmembrane	Sec-(SPI)
lp_0820	*glmM*	Phosphoglucosamine mutase	G: Carbohydrate transport and metabolism	-1,98	Intracellular	
lp_0828	_	hypothetical protein	_	1,81	Intracellular	
lp_0842	*ppk*	Polyphosphate kinase ATP-polyphosphate phosphotransferase Polyphosphoric acid kinase	P: Inorganic ion transport and metabolism	1,52	Intracellular	
lp_0843	*ppx3*	exopolyphosphatase	F: Nucleotide transport and metabolism	2,13	Intracellular	
lp_0848	_	purine transport protein	R: General function prediction only	-1,85	Intracellular	

lp_0856	_	acyltransferase	I: Lipid transport and metabolism	-2,09	Multi-transmembrane	Sec-(SPI)
lp_0858	_	redox protein, regulator of disulfide bond formation	O: Posttranslational modification, protein turnover, chaperones	1,66	Intracellular	
lp_0865	_	hypothetical protein	_	-2,35	Intracellular	
lp_0882	*glnM*	glutamine ABC transporter, permease protein	E: Amino acid transport and metabolism	1,59	Multi-transmembrane	Sec-(SPI)
lp_0899	_	membrane protein	_	-1,9	Multi-transmembrane	Sec-(SPI)
lp_0927	_	membrane protein	_	2,26	Multi-transmembrane	Sec-(SPI)
lp_0928	_	hypothetical protein	_	1,99	N-terminally anchored (No CS)	Sec-(SPI)
lp_0929	*asp1*	alkaline shock protein	S: Function unknown	1,84	Intracellular	
lp_0930	*asp2*	alkaline shock protein	S: Function unknown	1,98	Intracellular	
lp_0940	*hsdS1*	type I restriction-modification system,specificity subunit	V: Defense mechanisms	-2,03	Intracellular	
lp_0956	*asnC*	Asparagine--tRNA ligase 1 Asparaginyl-tRNA synthetase 1AsnRS 1	J: Translation, ribosomal structure and biogenesis	-1,85	Intracellular	
lp_0963	*rluE*	ribosomal large subunit pseudouridine synthase	J: Translation, ribosomal structure and biogenesis	-1,54	Intracellular	
lp_0994	_	no items found	_	-4,11	Intracellular	
lp_1005	*als*	acetolactate synthase	E: Amino acid transport and metabolism	1,63	Intracellular	
lp_1011	*dgk1*	deoxyadenosine kinase / deoxyguanosine kinase	F: Nucleotide transport and metabolism	1,82	Intracellular	
lp_1027	*fusA2*	Elongation factor G EF-G	J: Translation, ribosomal structure and biogenesis	-2,21	Intracellular	
lp_1054	*rpmD*	50S ribosomal protein L30	J: Translation, ribosomal structure and biogenesis	1,63	Intracellular	
lp_1063	*rplQ*	50S ribosomal protein L17	J: Translation, ribosomal structure and biogenesis	-2,35		
lp_1068	_	extracellular protein	S: Function unknown	-1,5	N-terminally anchored (No CS)	Sec-(SPI)
lp_1070	_	lipoprotein precursor, FMN-binding protein	S: Function unknown	-1,91	Lipid anchored	Sec-(SPII)

lp_1078	*rpsI*	30S ribosomal protein S9	J: Translation, ribosomal structure and biogenesis	-2,39	Intracellular	
lp_1096	*mtsB*	manganese ABC transporter, permease protein	P: Inorganic ion transport and metabolism	-1,87	Multi-transmembrane	Sec-(SPI)
lp_1097	*mtsA*	manganese/zinc ABC transporter, substrate binding protein	P: Inorganic ion transport and metabolism	-1,55	N-terminally anchored (No CS)	Sec-(SPI)
lp_1101	*ldh*	L-lactate dehydrogenase 2 L-LDH 2	C: Energy production and conversion	1,58	Intracellular	
lp_1116	*mleR1*	malolactic regulator, LysR family	K: Transcription	1,81	Intracellular	
lp_1120	_	amino acid transport protein	E: Amino acid transport and metabolism	-2,23	Multi-transmembrane	Sec-(SPI)
lp_1136	_	oxidoreductase, NAD(P)-dependent	R: General function prediction only	1,83	Intracellular	
lp_1150	_	diacylglycerol kinase family protein	R: General function prediction only	-1,64	Intracellular	
lp_1174	_	ribonuclease BN family protein	S: Function unknown	-1,85	Multi-transmembrane	Sec-(SPI)
lp_1190	*rfbD*	dTDP-4-dehydrorhamnose reductase	M: Cell wall/membrane/envelope biogenesis	-1,98	Intracellular	
lp_1215	*cps3A*	glycosyltransferase, family 2 (GT2)	R: General function prediction only	1,56	Intracellular	
lp_1229	_	mannose-specific adhesin, LPXTG-motif cell wall anchor	_	-1,83	LPxTG Cell-wall anchored	Sec-(SPI)
lp_1230	_	MarR family transcriptional regulator	K: Transcription	-1,95	Intracellular	
lp_1244	_	hypothetical protein	_	-1,88	Intracellular	
lp_1245	*hicD2*	L-2-hydroxyisocaproate dehydrogenase	C: Energy production and conversion	-1,75	Intracellular	
lp_1248	*tagD2*	glycerol-3-phosphate cytidylyltransferase	M: Cell wall/membrane/envelope biogenesis	-1,89	Intracellular	
lp_1259	_	anion exporter, TauE/SafE family	R: General function prediction only	1,73	Multi-transmembrane	Sec-(SPI)
lp_1280	*araT1*	aromatic amino acid specific aminotransferase	E: Amino acid transport and metabolism	-1,73	Intracellular	
lp_1292	_	GNAT family acetyltransferase	J: Translation, ribosomal structure and biogenesis	-2,5	Intracellular	

lp_1297	_	S-methylmethionine transport protein	E: Amino acid transport and metabolism	-1,51	Multi-transmembrane	Sec-(SPI)
lp_1300	_	membrane protein	_	-1,89	Multi-transmembrane	Sec-(SPI)
lp_1333	_	hypothetical protein	_	-1,58	Intracellular	
lp_1334	_	ABC transporter permease	_	-1,72	Multi-transmembrane	Sec-(SPI)
lp_1360	_	MarR family transcriptional regulator	K: Transcription	-1,79	Intracellular	
lp_1363	_	hypothetical protein	_	-1,53	Intracellular	
lp_1372	*gtcA1*	teichoic acid glycosylation protein	S: Function unknown	-1,71	Multi-transmembrane	Sec-(SPI)
lp_1388	_	hypothetical protein	_	-1,79	Intracellular	
lp_1394	_	hypothetical protein, N-terminus (pseudogene)	_	1,69	Intracellular	
lp_1406	*dltC2*	D-alanine--poly(phosphoribitol) ligase subunit 2-2 Full=D-alanyl carrier protein 2 DCP 2	I: Lipid transport and metabolism	-2,11	Intracellular	
lp_1427	_	nucleoside 2-deoxyribosyltransferase	F: Nucleotide transport and metabolism	-1,57	Intracellular	
lp_1436		riboflavin synthase, alpha chain		1,64		
lp_1437	*ribA*	3,4-dihydroxy-2-butanone 4-phosphate synthase /GTP cyclohydrolase II	H: Coenzyme transport and metabolism	1,95	Intracellular	
lp_1442	_	Crp/FNR family transcriptional regulator	T: Signal transduction mechanisms	-1,63	Intracellular	
lp_1445	*npr1*	NADH peroxidase	R: General function prediction only	-1,73	Intracellular	
lp_1468	_	ABC transporter, ATP-binding protein,iron-sulfur cluster assembly ATPase protein SufC	O: Posttranslational modification, protein turnover, chaperones	1,61	Intracellular	
lp_1483	_	Xre family transcriptional regulator	_	-1,67	Intracellular	
lp_1515	*infC*	Translation initiation factor IF-3	J: Translation, ribosomal structure and biogenesis	1,76	Intracellular	
lp_1517	*rplT*	50S ribosomal protein L20	J: Translation, ribosomal structure and biogenesis	-1,94	Intracellular	
lp_1544	*rrp5*	two-component system response regulator	T: Signal transduction mechanisms	-1,82	Intracellular	
lp_1545	*hpk5*	two-component system histidine protein kinase; sensor protein	T: Signal transduction mechanisms	-1,96	Multi-transmembrane	Sec-(SPI)
lp_1567	_	membrane protein	S: Function unknown	-1,7	Multi-transmembrane	Sec-(SPI)

lp_1573	*glk*	bifunctional protein: transcription regulator; sugar kinase, ROK family	K: Transcription	-2,18	Intracellular	
lp_1581	*glnA*	glutamate--ammonia ligase	E: Amino acid transport and metabolism	-2,73	Intracellular	
lp_1594	*rpmA*	50S ribosomal protein L27	J: Translation, ribosomal structure and biogenesis	-2,59	Intracellular	
lp_1613	*rpoZ*	DNA-directed RNA polymerase subunit omega	K: Transcription	-1,87	Intracellular	
lp_1622	_	thiamin pyrophosphokinase	H: Coenzyme transport and metabolism	-2,23	Intracellular	
lp_1626	_	phosphatase, dihydroxyacetone kinase family	R: General function prediction only	-2,02	Intracellular	
lp_1637	_	RNA binding protein	R: General function prediction only	-2,19	Intracellular	
lp_1640	*rplS*	50S ribosomal protein L19	J: Translation, ribosomal structure and biogenesis	-1,69	Intracellular	
lp_1687	_	Putative ribosome biogenesis GTPase RsgA 2 ribosome small subunit-dependent GTPase A	R: General function prediction only	-1,91	Intracellular	
lp_1702	_	membrane protein	S: Function unknown	-2,34	Multi-transmembrane	Sec-(SPI)
lp_1713	*lysA*	diaminopimelate decarboxylase	E: Amino acid transport and metabolism	-1,78	Intracellular	
lp_1722	_	4-aminobutanoate transport protein	E: Amino acid transport and metabolism	-1,72	Multi-transmembrane	Sec-(SPI)
lp_1732	*idi1*	isopentenyl diphosphate delta-isomerase	C: Energy production and conversion	-2,07	Intracellular	
lp_1742	_	hypothetical protein	_	-1,7	Intracellular	
lp_1757	_	ArsR family transcriptional regulator	K: Transcription	-1,99	Intracellular	
lp_1767	_	glycosyl hydrolase family protein	_	-1,85	Secretory(released) (with CS)	Sec-(SPI)
lp_1771	_	ABC transporter ATP-binding protein	P: Inorganic ion transport and metabolism	-1,79	Intracellular	
lp_1773	_	membrane protein	_	-2,33	Multi-transmembrane	Sec-(SPI)
lp_1778	*esbA*	membrane protein	_	1,7	Multi-transmembrane	Sec-(SPI)
lp_1787	*cat*	chloramphenicol O-acetyltransferase	V: Defense mechanisms	-1,87	Intracellular	
lp_1793	_	fibrinogen-binding family protein	K: Transcription	-1,64	Intracellular	

lp_1818	*tagB1*	ribitolphosphotransferase	M: Cell wall/membrane/envelope biogenesis	1,68	Intracellular	
lp_1836	*msrA3*	protein-methionine-S-oxide reductase	O: Posttranslational modification, protein turnover, chaperones	1,94	Intracellular	
lp_1869	*dfrA*	dihydrofolate reductase	H: Coenzyme transport and metabolism	1,71	Intracellular	
lp_1879	*hbsU*	DNA-binding protein	L: Replication, recombination and repair	-2,12	Intracellular	
lp_1881	*engA*	ribosome-associated GTPase	R: General function prediction only	-1,72	Intracellular	
lp_1887	_	riboflavin transporter	S: Function unknown	-2,24	Multi-transmembrane	Sec-(SPI)
lp_1898	*pfkA*	6-phosphofructokinase	G: Carbohydrate transport and metabolism	1,72	Intracellular	
lp_1914	_	MarR family transcriptional regulator	K: Transcription	1,53	Intracellular	
lp_1948	_	MarR family transcriptional regulator	K: Transcription	1,81	Intracellular	
lp_1953	_	hypothetical protein	_	-1,71	Intracellular	
lp_1956	_	ABC transporter permease	_	-2,01	Multi-transmembrane	Sec-(SPI)
lp_1972	_	hypothetical protein	S: Function unknown	-2,09	Intracellular	
lp_1982	*lytH*	N-acetylmuramoyl-L-alanine amidase	M: Cell wall/membrane/envelope biogenesis	-2,14		
lp_1985	_	phosphohydrolase	R: General function prediction only	-1,91	Intracellular	
lp_1988	_	membrane protein	_	-1,82	Multi-transmembrane	Sec-(SPI)
lp_1997	_	integrase, fragment	L: Replication, recombination and repair	-1,54	Intracellular	
lp_2001	_	membrane protein	_	-2,77	Multi-transmembrane	Sec-(SPI)
lp_2039	*rbfA*	ribosome-binding factor A	J: Translation, ribosomal structure and biogenesis	-2,19	Intracellular	
lp_2056	_	HAD-superfamily hydrolase	R: General function prediction only	-1,74	Intracellular	
lp_2057	*ldhD*	D-lactate dehydrogenase D-LDH	C: Energy production and conversion	-2,77	Intracellular	

lp_2066	_	hypothetical protein	_	-2,03	Multi-transmembrane	Sec-(SPI)
lp_2086	*apt*	Adenine phosphoribosyltransferase APRT	F: Nucleotide transport and metabolism	-1,71	Intracellular	
lp_2105	*galE3*	UDP N-acetyl glucosamine 4-epimerase, NAD dependent	M: Cell wall/membrane/envelope biogenesis	1,67	Intracellular	
lp_2108	*cps4A*	polysaccharide biosynthesis protein, chain length regulator	M: Cell wall/membrane/envelope biogenesis	2,07	Multi-transmembrane	Sec-(SPI)
lp_2110	*glnQ3*	glutamine ABC transporter ATP-binding protein	E: Amino acid transport and metabolism	-1,7	Intracellular	
lp_2114	_	NTP pyrophosphohydrolase	R: General function prediction only	-2,72	Intracellular	
lp_2122	_	metallo-beta-lactamase superfamily protein	R: General function prediction only	-2,17	Intracellular	
lp_2125	*rpsO*	30S ribosomal protein S15	J: Translation, ribosomal structure and biogenesis	-2,64	Intracellular	
lp_2126	*rpsT*	30S ribosomal protein S20	J: Translation, ribosomal structure and biogenesis	-2,48	Intracellular	
lp_2133	*coaD*	pantetheine-phosphate adenylyltransferase	H: Coenzyme transport and metabolism	1,84	Intracellular	
lp_2162	_	extracellular protein, NlpC/P60 family,gamma-D-glutamate-meso-diaminopimelate muropeptidase	M: Cell wall/membrane/envelope biogenesis	-2,04	N-terminally anchored (No CS)	Sec-(SPI)
lp_2199	*mraY*	Phospho-N-acetylmuramoyl-pentapeptide-transferase UDP-MurNAc-pentapeptide phosphotransferase	M: Cell wall/membrane/envelope biogenesis	-2,30	Multi-transmembrane	Sec-(SPI)
lp_2201	*ftsL*	cell division protein FtsL	D: Cell cycle control, cell division, chromosome partitioning	-1,53	N-terminally anchored (No CS)	Sec-(SPI)
lp_2215	*rrmA*	rRNA large subunit methyltransferase A	H: Coenzyme transport and metabolism	-1,67	Intracellular	
lp_2229	_	beta-lactamase superfamily metal-dependent hydrolase	R: General function prediction only	-2,02	Intracellular	
lp_2230	_	hypothetical protein	_	-1,73	Intracellular	

lp_2240	_	amino acid transport protein	E: Amino acid transport and metabolism	-2,38	Multi-transmembrane	Sec-(SPI)
lp_2270	*trxA2*	thioredoxin	O: Posttranslational modification, protein turnover, chaperones	-1,81	Intracellular	
lp_2273	_	hypothetical protein UPF0473	S: Function unknown	-1,71	Intracellular	
lp_2277	*alaS*	Alanine--tRNA ligase Alanyl-tRNA synthetase AlaRS	J: Translation, ribosomal structure and biogenesis	-1,75	Intracellular	
lp_2278	*rhe3*	ATP-dependent RNA helicase	L: Replication, recombination and repair	-1,58	Intracellular	
lp_2281	_	preprotein translocase subunit YajC	U: Intracellular trafficking, secretion, and vesicular transport	-1,79	N-terminally anchored (No CS)	Sec-(SPI)
lp_2308	*thrA2*	aspartate kinase	E: Amino acid transport and metabolism	-1,8	Intracellular	
lp_2317	*mreD*	cell shape determining protein MreD	M: Cell wall/membrane/envelope biogenesis	-1,61	Multi-transmembrane	Sec-(SPI)
lp_2334	_	membrane protein	_	-2,12	Multi-transmembrane	Sec-(SPI)
lp_2341	*bla2*	beta-lactamase, class A	V: Defense mechanisms	-1,61	N-terminally anchored (No CS)	Sec-(SPI)
lp_2342	_	Xre family transcriptional regulator	_	-2,09	Intracellular	
lp_2346	_	alcohol dehydrogenase	C: Energy production and conversion	1,49	Intracellular	
lp_2347	_	hypothetical protein	_	1,55	Intracellular	
lp_2365	*atpG*	ATP synthase gamma chain ATP synthase F1 sector gamma subunit F-ATPase gamma subunit	C: Energy production and conversion	1,78	Intracellular	
lp_2367	*atpH*	ATP synthase subunit delta ATP synthase F(1) sector subunit delta F-type ATPase subunit delta F-ATPase subunit delta	C: Energy production and conversion	1,60	Intracellular	
lp_2368	*atpF*	ATP synthase subunit b ATP synthase F(0) sector subunit b ATPase subunit I F-type ATPase subunit b F-ATPase subunit b	C: Energy production and conversion	1,55	N-terminally anchored (No CS)	Sec-(SPI)
lp_2378	*prfA*	peptide chain release factor 1	J: Translation, ribosomal structure and biogenesis	-1,7	Intracellular	

lp_2390	*bcaT*	branched-chain amino acid aminotransferase	E: Amino acid transport and metabolism	-1,96	Intracellular	
lp_2443	_	prophage P2a protein 14	S: Function unknown	-1,53	Intracellular	
lp_2454	_	prophage P2a protein 3; DNA adenine methylase	L: Replication, recombination and repair	-1,97	Intracellular	
lp_2502	*pgi*	glucose-6-phosphate isomerase	G: Carbohydrate transport and metabolism	-2,05	Intracellular	
lp_2516	_	hypothetical protein	_	-2,58	Intracellular	
lp_2525	_	ABC transporter ATP-binding protein	R: General function prediction only	-3,27	Intracellular	
lp_2531	*pts18CBA*	PTS system, N-acetylglucosamine and glucose-specific EIICBA component	G: Carbohydrate transport and metabolism	-2,29	Multi-transmembrane	Sec-(SPI)
lp_2544	*npr2*	NADH peroxidase	R: General function prediction only	1,86	Intracellular	
lp_2580	_	sulfatase, alkaline phosphatase superfamily,membrane-bound	M: Cell wall/membrane/envelope biogenesis	-1,72	Multi-transmembrane	Sec-(SPI)
lp_2596	*pflA1*	formate acetyltransferase activating enzyme	O: Posttranslational modification, protein turnover, chaperones	1,81	Intracellular	
lp_2613	_	ABC transporter permease	_	-3,42	Multi-transmembrane	Sec-(SPI)
lp_2614	_	ABC transporter ATP-binding protein	V: Defense mechanisms	-2,06	Intracellular	
lp_2615	_	GntR family transcriptional regulator	K: Transcription	-2,04	Intracellular	
lp_2631	_	lipase/esterase	E: Amino acid transport and metabolism	-1,84	Intracellular	
lp_2642	_	membrane protein	_	1,55	Multi-transmembrane	Sec-(SPI)
lp_2647	*pts19A*	PTS system,N-acetylglucosamine/galactosamine-specific EIIA component	G: Carbohydrate transport and metabolism	-2,04	Intracellular	
lp_2659	*xpk1*	xylulose-5-P phosphoketolase / fructose-6-P phosphoketolase	G: Carbohydrate transport and metabolism	1,69	Intracellular	
lp_2664	_	metal-dependent phosphohydrolase, HD family	R: General function prediction only	1,68	Intracellular	
lp_2710	*pucK*	xanthine permease	F: Nucleotide transport and metabolism	-2,48	Multi-transmembrane	Sec-(SPI)

lp_2716	*ica3*	family 2 glycosyltransferase	M: Cell wall/membrane/envelope biogenesis	-1,58	Multi-transmembrane	Sec-(SPI)
lp_2727	*purC*	Phosphoribosylaminoimidazole-succinocarboxamide synthase SAICAR synthetase	F: Nucleotide transport and metabolism	1,62	Intracellular	
lp_2735	*ptp3*	protein-tyrosine phosphatase	T: Signal transduction mechanisms	1,71	Intracellular	
lp_2738	_	L-asparaginase	E: Amino acid transport and metabolism	1,535	Intracellular	
lp_2739	_	ABC transporter ATP-binding protein	V: Defense mechanisms	-4,73	Intracellular	
lp_2740	_	ABC transporter permease	Q: Secondary metabolites biosynthesis, transport and catabolism	-3,81	Multi-transmembrane	Sec-(SPI)
lp_2741	_	membrane protein	_	-1,56	Multi-transmembrane	Sec-(SPI)
lp_2742	_	GntR family transcriptional regulator	K: Transcription	-1,84	Intracellular	
lp_2743	_	ABC transporter ATP-binding protein	V: Defense mechanisms	-1,54	Intracellular	
lp_2744	_	ABC transporter permease	_	-3,03	Multi-transmembrane	Sec-(SPI)
lp_2765	_	hypothetical protein	_	1,51	N-terminally anchored (No CS)	Sec-(SPI)
lp_2776	*dsdA*	Probable D-serine dehydratase D-serine deaminase DSD	E: Amino acid transport and metabolism	-2,57	Intracellular	
lp_2783	_	family 2 glycosyltransferase	M: Cell wall/membrane/envelope biogenesis	-2,04	Intracellular	
lp_2804	_	LysR family transcriptional regulator	K: Transcription	1,77	Intracellular	
lp_2812	_	extracellular protein, membrane-anchored	S: Function unknown	-2,13	N-terminally anchored (No CS)	Sec-(SPI)
lp_2818	_	proton/sodium-glutamate/aspartate symport protein	C: Energy production and conversion	-1,75	Multi-transmembrane	Sec-(SPI)
lp_2823	_	ABC transporter ATP-binding protein	V: Defense mechanisms	-1,78	Intracellular	
lp_2827	*napA3*	Na(+)/H(+) antiporter	P: Inorganic ion transport and metabolism	-3,51	Multi-transmembrane	Sec-(SPI)
lp_2829	*mleP3*	malate transport protein	R: General function prediction only	-1,85	Multi-transmembrane	Sec-(SPI)

lp_2830	*aspA*	aspartate ammonia-lyase	E: Amino acid transport and metabolism	-1,68	Intracellular	
lp_2840	_	ribosomal protein serine-acetylating enzyme	J: Translation, ribosomal structure and biogenesis	-1,5	Intracellular	
lp_2844	*tagE6*	poly(glycerol-phosphate) alpha-glucosyltransferase	M: Cell wall/membrane/envelope biogenesis	1,6	Intracellular	
lp_2863	*sip2*	signal peptidase I	U: Intracellular trafficking, secretion, and vesicular transport	-1,56	N-terminally anchored (No CS)	Sec-(SPI)
lp_2888	*patB*	cystathionine beta-lyase	E: Amino acid transport and metabolism	1,6	Intracellular	
lp_2907	_	GNAT family acetyltransferase	J: Translation, ribosomal structure and biogenesis	1,56	Intracellular	
lp_2912	_	ABC transporter ATP-binding protein	R: General function prediction only	-2,22	Intracellular	
lp_2918	*ropB*	TetR family transcriptional regulator	K: Transcription	-1,62	Intracellular	
lp_2932	*nrdD*	anaerobic ribonucleoside-triphosphate reductase	F: Nucleotide transport and metabolism	1,742	Intracellular	
lp_2949	_	membrane protein	_	-1,82	Multi-transmembrane	Sec-(SPI)
lp_2953	_	esterase	R: General function prediction only	1,68	Intracellular	
lp_2960		lipase/esterase, subfamily of SGNH-hydrolases		1,68		
lp_2992	*mntH2*	manganese transport protein	P: Inorganic ion transport and metabolism	-2,08	Multi-transmembrane	Sec-(SPI)
lp_2993	_	nucleotide-binding protein, universal stress protein UspA family	T: Signal transduction mechanisms	-2,53	Intracellular	
lp_3015	_	extracellular transglycosylase, with LysM peptidoglycan binding domain	M: Cell wall/membrane/envelope biogenesis	-1,66	Secretory(released) (with CS)	Sec-(SPI)
lp_3019	_	extracellular protein, membrane-anchored	_	-1,54	N-terminally anchored (No CS)	Sec-(SPI)
lp_3040	_	multidrug ABC transporter, ATP-binding and permease protein	V: Defense mechanisms	1,88	Multi-transmembrane	Sec-(SPI)
lp_3045	_	short-chain dehydrogenase/oxidoreductase,classical SDR family, subgroup 1	I: Lipid transport and metabolism	-1,58	Intracellular	

lp_3059	_	adherence-associated mucus-binding protein,LPXTG-motif cell wall anchor	_	1,76	LPxTG Cell-wall anchored	Sec-(SPI)
lp_3060	_	AraC family transcriptional regulator	K: Transcription	1,97	Intracellular	
lp_3085	*asnB2*	asparagine synthase	E: Amino acid transport and metabolism	-2,82	Intracellular	
lp_3087	*rrp10*	two-component system response regulator	K: Transcription	-1,61	Intracellular	
lp_3096	_	short-chain dehydrogenase	R: General function prediction only	-1,54	N-terminally anchored (No CS)	Sec-(SPI)
lp_3169	_	hypothetical protein	_	-2,31	Intracellular	
lp_3173	_	cell surface protein, membrane-anchored	_	-1,54		
lp_3174	*cfa2*	cyclopropane-fatty-acyl-phospholipid synthase	M: Cell wall/membrane/envelope biogenesis	-1,58	Intracellular	
lp_3179	_	hypothetical protein	_	-1,67	Intracellular	
lp_3180	_	membrane protein	_	-2,2	Multi-transmembrane	Sec-(SPI)
lp_3183	_	sulfite export protein	_	-1,94	Multi-transmembrane	Sec-(SPI)
lp_3184	_	branched-chain amino acid transport protein	S: Function unknown	-3,38	Multi-transmembrane	Sec-(SPI)
lp_3211	_	cystine ABC transporter ATP-binding protein	E: Amino acid transport and metabolism	-1,95	Intracellular	
lp_3214	_	cystathionine ABC transporter, substrate binding protein	E: Amino acid transport and metabolism	-1,59	Lipid anchored	Sec-(SPII)
lp_3227	_	hypothetical protein	_	-2,02	Intracellular	
lp_3244	_	NADPH-dependent FMN reductase family protein	R: General function prediction only	1,52	Intracellular	
lp_3248	_	bacteriocin immunity protein	_	-1,81	Intracellular	
lp_3254	*lrgA*	murein hydrolase export protein	R: General function prediction only	-3,81	Multi-transmembrane	Sec-(SPI)
lp_3255	*lrgB*	murein hydrolase regulator	M: Cell wall/membrane/envelope biogenesis	-2,74	Multi-transmembrane	Sec-(SPI)
lp_3256	_	DegV family protein	S: Function unknown	-1,75	Intracellular	
lp_3267	*gshR4*	glutathione reductase	C: Energy production and conversion	1,82	Intracellular	
lp_3269	*purB*	adenylosuccinate lyase	F: Nucleotide transport and metabolism	-1,78	Intracellular	

lp_3286	*hly*	membrane protein	R: General function prediction only	-1,84	Multi-transmembrane	Sec-(SPI)
lp_3288	_	cation (cobalt-zinc-cadmium) efflux protein	P: Inorganic ion transport and metabolism	-1,59	Multi-transmembrane	Sec-(SPI)
lp_3292	_	hypothetical protein	_	-2,25	Multi-transmembrane	Sec-(SPI)
lp_3312	_	beta-lactamase family protein	V: Defense mechanisms	1,55	Intracellular	
lp_3314	*pflA2*	formate C-acetyltransferase activating enzyme	O: Posttranslational modification, protein turnover, chaperones	-1,53	Intracellular	
lp_3324	_	glycine betaine/carnitine/choline transport protein	M: Cell wall/membrane/envelope biogenesis	-3,38	Multi-transmembrane	Sec-(SPI)
lp_3334	*adeC*	Adenine deaminase Adenase Adenine aminase	F: Nucleotide transport and metabolism	-2,48	Intracellular	
lp_3348	_	hypothetical protein	_	-1,79	Intracellular	
lp_3355	_	short-chain dehydrogenase/oxidoreductase,atypical SDR family, subgroup 6	M: Cell wall/membrane/envelope biogenesis	1,75	Intracellular	
lp_3356	_	GNAT family acetyltransferase	R: General function prediction only	1,7	Intracellular	
lp_3378	_	prophage P3 protein 12	_	-1,71	Intracellular	
lp_3382	_	prophage P3 protein 8, DNA primase/helicase	R: General function prediction only	-1,85	Intracellular	
lp_3393	_	cell surface hydrolase, membrane-bound	R: General function prediction only	1,70	N-terminally anchored (No CS)	Sec-(SPI)
lp_3394	_	hypothetical protein	_	1,76	Intracellular	
lp_3397	_	nucleoside 2-deoxyribosyltransferase	F: Nucleotide transport and metabolism	1,59	Intracellular	
lp_3412	_	cell surface protein, CscB family	_	-1,91	N-terminally anchored (No CS)	Sec-(SPI)
lp_3430	_	peroxidase	P: Inorganic ion transport and metabolism	-1,91	Intracellular	
lp_3531	_	LacI family transcriptional regulator	K: Transcription	-1,65	Intracellular	
lp_3552	_	hypothetical protein	J: Translation, ribosomal structure and biogenesis	-1,62	Intracellular	

lp_3558	*araR*	GntR family transcriptional regulator	K: Transcription	-1,67	Intracellular	
lp_3578	*kat*	catalase	P: Inorganic ion transport and metabolism	1,70	Intracellular	
lp_3579	*nrpR5*	RNA polymerase (RNAP)-binding regulatory protein, arsenate reductase (ArsC) family, Spx subfamily	P: Inorganic ion transport and metabolism	-2,04	Intracellular	
lp_3587	*pox4*	pyruvate oxidase	E: Amino acid transport and metabolism	1,62	Intracellular	
lp_3665	*pdc*	phenolic acid decarboxylase	Q: Secondary metabolites biosynthesis, transport and catabolism	1,88	Intracellular	
lp_3673	*pepC2*	aminopeptidase C	E: Amino acid transport and metabolism	1,52	Intracellular	
lp_3676	_	cell surface protein precursor, CscC family	_	-1,84	Secretory(released) (with CS)	Sec-(SPI)
lp_3687	_	Membrane protein insertase YidC 2 Foldase YidC 2 Membrane integrase YidC 2 Membrane protein YidC 2 Precursor	U: Intracellular trafficking, secretion, and vesicular transport	-1,67	Multi-transmembrane(Lipid modified N-termini	Sec-(SPII)

[a] Fold change refers to incubation in MRS supplemented with hydroxytyrosol 10 mM relative to growth in MRS lacking HXT. [b] FDR ≤ 0.05. [c] LocateP DataBase (http://bamics2.cmbi.ru.nl/websoftware/locatep2/locatep2_start.php) CS: CleavageSite; Sec-(SPI): Secretory Pathway I; Sec-(SPII): Secretory Pathway II.
